# Supplementary material for: Individual retrotransposon integrants are differentially controlled by KZFP/KAP1-dependent histone methylation, DNA methylation and TET-mediated hydroxymethylation in naïve embryonic stem cells
Source: Epigenetics Chromatin. 2018 Feb 26;11:7. doi: 10.1186/s13072-018-0177-1 (PMC6389204; doi:10.1186/s13072-018-0177-1)
Supplement: Supplementary file 11 — Additional file 11. Pattern analysis. [file 13072_2018_177_MOESM11_ESM.zip › Patterns analysis/DataTables/extensions/Responsive/examples/display-control/index.html]

Responsive examples - Display control


# Responsive example Display control

Responsive has two basic modes of operation for controlling the visibility of columns at different
display sizes. These two modes can be using either separately or together:

- Manually assigned class names for breakpoints - Assign a column a class name to tell Responsive
  which breakpoint(s) to show it in.
- Automatically - for columns without a breakpoint class name, it will be automatically removed
  if there is no room available on screen to show it. Columns are removed from the right, moving
  left.

This section explores these two options.

### Display control

- Automatic column hiding
- Class control
- Assigned class control

Please refer to the DataTables documentation for full
information about its API properties and methods.  
Additionally, there are a wide range of extras and
plug-ins which extend the capabilities of
DataTables.

DataTables designed and created by SpryMedia Ltd © 2007-2014  
DataTables is licensed under the MIT license.
